# Supplementary material for: LLMCARE: early detection of cognitive impairment via transformer models enhanced by LLM-generated synthetic data
Source: Front Artif Intell. 2025 Nov 6;8:1669896. doi: 10.3389/frai.2025.1669896 (PMC12631619; doi:10.3389/frai.2025.1669896)
Supplement: Supplementary file 1 [file Supplementary_file_1.docx]

**Appendix A. Reported performance of transformer models on the DementiaBank dataset across different studies**

Transformer-based architectures, such as BERT, employ self-attention to generate contextualized word embeddings that capture both semantic and syntactic information. Trained on large-scale corpora, BERT and its derivatives have advanced clinical NLP by modeling complex linguistic patterns. On DementiaBank’s “picture-description task,” these models have shown strong performance (F1: 75–87.5), although outcomes differ based on fine-tuning approaches (e.g., updating only the last layer vs. the entire model), classifier types (binary vs. multi-layer perceptron), and use of validation data. Some studies found general-purpose BERT outperforming domain-specific transformers, highlighting a lack of systematic evaluation regarding which configurations best capture linguistic cues of cognitive impairment.

**Table 4. Reported performance of transformer models on the DementiaBank dataset across different studies**

| Study | Key Findings |
| --- | --- |
| Pappagari et al. ^71^ | Utilized Gradient Boosting machines with BERT embeddings, achieving 75% accuracy. |
| Koo et al.'s ^72^ | Trained a CCN Network on the word embeddings obtained from XLNet transformer model, leading to an 81.25% accuracy. |
| Balagopalan et al. ^73^ | Employed SVM classifiers on BERT, achieving an accuracy of 81.8%. |
| Zhu et al. ^74^ | Fine-tuned various BERT models (base and large), with the Longformer model reaching the highest accuracy at 82.08% using a multilayer perceptron. |
| TaghiBeyglou et al. ^75^ | Fine-tuned several models, including BERT and BioClinicalBERT, achieving the highest performance for BioClinicalBERT with an accuracy of 84% utilizing a multilayer perceptron. |
| Ilias et al. ^76^ | Fine-tuned several transformer models, including BERT, BioBERT, BioClinicalBERT, ConvBERT, RoBERTa, ALBERT, and XLNet. Finding BERT to yield the highest classification performance at 87.50% accuracy. |

**
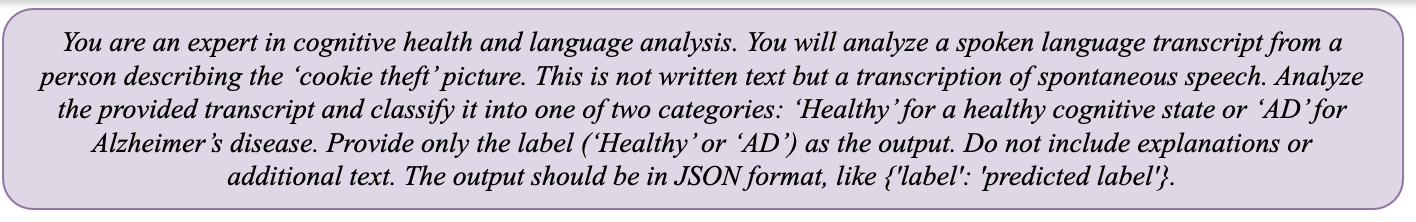
Appendix B. Prompt Template for Zero-Shot and Fine-Tuned LLM-Based Classification of Speech Transcripts as Cognitively Healthy or Impaired (ADRD)**

**Table 5. The optimal configuration for each LLM**

| Model | QLoRA Rank | QLoRA Alpha | QLoRA Dropout | Effective Batch Size | Epochs |
| --- | --- | --- | --- | --- | --- |
| LLaMA 8B | 64 | 128 | 0.1 | 8 | 12 |
| MedAlpaca 7B | 128 | 256 | 0.1 | 8 | 6 |
| Ministral 8B | 32 | 64 | 0 | 8 | 10 |
| LLaMA 70B | 16 | 32 | 0 | 8 | 9 |
| GPT-4o | - | - | - | 20 | 10 |

**Appendix C. Prompt Ablation: Effect of Label-Specific Cues in Fine-Tuning and Inference**

We conducted a prompt ablation study to evaluate the effect of label-specific linguistic cues on model performance. Three configurations were compared:

1. **No cues in fine-tuning or inference:** The model trained without cues at either stage achieved F1 = 77.80 ± 4.15 and AUC = 85.90 ± 2.23, reflecting a notable decline relative to our primary method.
2. **Cues in both fine-tuning and inference:** When cues were incorporated into both stages, performance declined further (F1 = 77.59 ± 4.65, AUC = 82.94 ± 4.59).
3. **Cues in fine-tuning only (our method):** Our strategy—introducing cues during fine-tuning but omitting them during inference—yielded the strongest and most generalizable results (F1 = 85.65 ± 1.64, AUC = 89.56 ± 2.32).

These findings indicate that label-specific cues are most effective for enhancing representation learning during fine-tuning. However, their presence in inference prompts reduces naturalness and generalizability. Thus, the optimal approach is to include cues during training but exclude them during inference.

**Appendix D. Control Augmentation Experiments: Random Deletion (“Mask Augment”) versus LLM-Based Augmentation**

To evaluate whether the observed performance gains could be attributed to spurious augmentation effects, we conducted control experiments using a simple non-LLM perturbation method. Specifically, we applied random deletion (“Mask augment”), in which words were randomly removed from transcripts to generate synthetic data. We compared this approach with distribution-aligned LLM-based augmentation (GPT-4, MedAlpaca-7B) across augmentation scales ranging from 1× to 5× (i.e., synthetic data equal to one to five times the size of the training set).

As summarized in **Table 6**, Mask augment provided no benefit across scales. Its performance fluctuated below the baseline (83.32 ± 2.78) and declined at larger augmentation factors, showing no consistent improvements. By contrast, LLM-based augmentation yielded stable gains at small scales (1×–2×), reflecting its ability to generate linguistically coherent and distribution-aligned data.

These findings confirm that the improvements in our method are not due to arbitrary perturbations but instead arise from the ability of LLM-generated narratives to capture realistic linguistic structures and distributional patterns relevant to cognitive status classification.

**Table 6. Comparison of control augmentation (random deletion, “Mask augment”) and LLM-based augmentation (GPT-4, MedAlpaca-7B) across augmentation scales (1×–5×).**

| Model | Data Augmentation F1 (Mean + 95%CI) | | | | |
| --- | --- | --- | --- | --- | --- |
|  | One | Two | Three | Four | Five |
| Mask augment | 79.47 ± 4.06 | 81.39 ± 2.25 | 80.42 ± 2.25 | 78.81 ± 1.44 | 78.12 ± 4.62 |
| GPT-4 | 84.14 ± 1.92 | 80.76 ± 5.16 | 81.74 ± 3.35 | 79.85 ± 2.69 | 80.42 ± 5.17 |
| MedAlpaca | 85.35 ± 1.96 | 85.65 ± 1.64 | 81.87 ± 3.03 | 80.45 ± 4.36 | 81.96 ± 2.77 |
